# Use of capillary blood glucose for screening for gestational diabetes mellitus in resource-constrained settings

**DOI:** 10.1007/s00592-015-0761-9

**Published:** 2015-04-28

**Authors:** Balaji Bhavadharini, Manni Mohanraj Mahalakshmi, Kumar Maheswari, Gunasekaran Kalaiyarasi, Ranjit Mohan Anjana, Mohan Deepa, Harish Ranjani, Miranda Priya, Ram Uma, Sriram Usha, Sonak D. Pastakia, Belma Malanda, Anne Belton, Ranjit Unnikrishnan, Arivudainambi Kayal, Viswanathan Mohan

**Affiliations:** Madras Diabetes Research Foundation and Dr. Mohan’s Diabetes Specialities Centre, WHO Collaborating Centre for Non-communicable Diseases Prevention and Control, IDF Centre of Education, 4, Conran Smith Road, Gopalapuram, Chennai, 600 086 India; Seethapathy Clinic and Hospital, Chennai, India; Associates in Clinical Endocrinology Education and Research (ACEER), Chennai, India; College of Pharmacy, Purdue University, West Lafayette, IN USA; International Diabetes Federation, Brussels, Belgium

**Keywords:** Gestational diabetes mellitus, Capillary blood glucose, Venous plasma glucose, CBG, WHO 1999 criteria, IADPSG criteria, Screening, Asian Indian, South Asians

## Abstract

**Aims:**

The aim of the study was to evaluate usefulness of capillary blood glucose (CBG) for diagnosis of gestational diabetes mellitus (GDM) in resource-constrained settings where venous plasma glucose (VPG) estimations may be impossible.

**Methods:**

Consecutive pregnant women (*n* = 1031) attending antenatal clinics in southern India underwent 75-g oral glucose tolerance test (OGTT). Fasting, 1- and 2-h VPG (AU2700 Beckman, Fullerton, CA) and CBG (One Touch Ultra-II, LifeScan) were simultaneously measured. Sensitivity and specificity were estimated for different CBG cut points using the International Association of Diabetes in Pregnancy Study Groups (IADPSG) criteria for the diagnosis of GDM as gold standard. Bland–Altman plots were drawn to look at the agreement between CBG and VPG. Correlation and regression equation analysis were also derived for CBG values.

**Results:**

Pearson’s correlation between VPG and CBG for fasting was *r* = 0.433 [intraclass correlation coefficient (ICC) = 0.596, *p* < 0.001], for 1H, it was *r* = 0.653 (ICC = 0.776, *p* < 0.001), and for 2H, *r* = 0.784 (ICC = 0.834, *p* < 0.001). Comparing a single CBG 2-h cut point of 140 mg/dl (7.8 mmol/l) with the IADPSG criteria, the sensitivity and specificity were 62.3 and 80.7 %, respectively. If CBG cut points of 120 mg/dl (6.6 mmol/l) or 110 mg/dl (6.1 mmol/l) were used, the sensitivity improves to 78.3 and 92.5 %, respectively.

**Conclusions:**

In settings where VPG estimations are not possible, CBG can be used as an initial screening test for GDM, using lower 2H CBG cut points to maximize the sensitivity. Those who screen positive can be referred to higher centers for definitive testing, using VPG.

**Electronic supplementary material:**

The online version of this article (doi:10.1007/s00592-015-0761-9) contains supplementary material, which is available to authorized users.

## Introduction

The prevalence of gestational diabetes mellitus (GDM) is rapidly increasing and currently affects up to 15 % of pregnant women worldwide [1]. In India, in 2011, 62.4 million people were reported to have diabetes [2], while 4 million women were reported to have GDM [3].

Screening and diagnosis of GDM has been a matter of intense debate. The oral glucose tolerance test (OGTT) remains the gold standard for diagnosis of GDM. Based on the Hyperglycemia and Adverse Pregnancy Outcome (HAPO) study [4], the International Association of Diabetes in Pregnancy Study Groups (IADPSG) criteria were developed which recommends three venous plasma glucose samples, i.e., fasting, one and 2 h after administration of 75 g glucose [5]. However, in many resource-constrained settings in the developing world, obtaining venous samples may be difficult or indeed impossible, due to shortage of trained phlebotomists and limited access to standardized laboratories. In such situations, if screening for GDM is to be done at all, the only alternative would be to use a handheld blood glucose meter to perform capillary blood glucose (CBG) testing. Currently, the use of CBG for diagnosis of GDM is not recommended. There are few studies comparing CBG with the old WHO 1999 criteria for GDM [6] but none, to our knowledge, have compared CBG with IADPSG criteria.

The objectives of this paper, therefore, were as follows:

1. To compare capillary blood glucose (CBG) estimation using a handheld glucose meter with the venous plasma glucose (VPG) estimation using the IADPSG criteria as the gold standard, for diagnosis of GDM.
2. To derive regression equations for the VPG fasting, 1- and 2-h values from the corresponding CBG estimations and
3. To see whether CBG can at least be used as an initial screening test before referring patients to higher centers for a diagnostic OGTT using VPG.

## Methods

This study is a part of an ongoing program called Women in India with GDM Strategy (WINGS) conducted under the auspices of the International Diabetes Federation (IDF) [7]. One of the aims of the WINGS program was to identify the best strategy which could be widely applied in resource-constrained settings for screening of women with GDM. This paper deals with the results of the screening study, using CBG and VPG carried out between January and November 2013.

### Ethical clearance

All participants gave written informed consent prior to participating in the study. All procedures followed were in accordance with the ethical standards and in keeping with the Helsinki Declaration of 1975, as revised in 2008. Permission was obtained from the Director of Public Health and the Health Secretary, Government of Tamil Nadu to undertake the WINGS program. The study proposal was approved by the Institutional Ethics Committee of the Madras Diabetes Research Foundation, Chennai, India.

### Study sites

Nine urban health centers in Chennai city and 11 rural centers in Kanchipuram district, from Tamil Nadu state in southern India, were selected for this study.

### Pilot screening method

All participants gave written informed consent to participate in the study. For all participants, an interviewer-administered case report form was used to obtain demographic information including age and period of gestation (weeks), along with general medical history and family history of diabetes. Anthropometric measurements were done using standard techniques. Height was measured using a stadiometer (SECA Model 213, Seca Gmbh Co, Hamburg, Germany) to the nearest 0.1 cm, and weight was measured with an electronic weighing machine (SECA Model 803, Seca Gmbh Co) to the nearest 0.1 kg. The body mass index (BMI) was calculated using the formula weight (in kg) divided by height in meters (squared) [8]. All participants underwent a 75-g oral glucose tolerance test (OGTT) in the fasting state, i.e., after at least 8 h of no caloric intake. Venous plasma glucose (VPG) and capillary blood glucose (CBG) were measured simultaneously (within 1–2 min of each other).

Blood samples were collected in sodium fluoride/Na2 EDTA Vacutainer tubes to prevent glycolysis. Samples were transported to the central laboratory within 1–2 h in cool boxes which had gel packs to maintain the temperature between 2 and 8 °C.

Fasting, 1-h (1H) and 2-h (2H) samples were drawn from the antecubital vein, and VPG was measured in our laboratory using an autoanalyzer (AU2700 Beckman, Fullerton, CA). Capillary blood samples were also simultaneously drawn at fasting, 1 and 2 h from a finger stick blood sample, and CBG was measured using a handheld glucose meter (One Touch Ultra-II, LifeScan, Johnson & Johnson, Milpitas, CA). The glucose meter used is plasma calibrated, and without hematocrit correction, it provides accurate results for a hematocrit range of 30–50 %.The intra- and inter-assay coefficients of variation (CV) for the venous blood glucose ranged from 0.78 to 1.68 %, while the mean coefficient of variation for CBG was 4.2 %. Hemoglobin was measured using SLS (sodium lauryl sulfate)–hemoglobin method.

### Definitions of GDM

1. Using VPG: According to the IADPSG criteria [5], GDM was diagnosed based on any one of the following VPG values obtained in the OGTT: fasting ≥ 92 mg/dl (5.1 mmol/l), 1 h ≥ 180 mg/dl (9.9 mmol/l) or 2 h ≥ 153 mg/dl (8.4 mmol/l).
2. Using CBG: There are no defined cut points for CBG in pregnancy. To compare with the IADPSG criteria, we used the same CBG cut points as IADPSG criteria VPG values given above. In non-pregnant adults, the World Health Organization (WHO) recommends using 20 mg/dl higher values for post-glucose load CBG estimations [9]. Hence, additionally, we also used 20 mg/dl (1.1 mmol/l) higher CBG cut points for the 1- and 2-h values [i.e., 200 mg/dl (11.1 mmol/l) and 173 mg/dl (9.6 mmol/l)].

### Statistical analysis

Statistical analyses were performed using SPSS for Windows version 20 (SPSS Inc., Chicago, IL). Sensitivity, specificity, positive predictive value, negative predictive value and accuracy of different CBG cut points were calculated for the IADPSG criteria using MedCalc version 12.7.0. Bland–Altman plots were drawn to look at the agreement between CBG and VPG in fasting, 1H and 2H samples and to see whether it was within the 95 % limits. Pearson’s correlation was used to compare CBG and VPG values at fasting, 1H and 2H time points. Regression equations were derived for VPG values at fasting, 1H and 2H using the CBG estimations.

## Results

The mean age of the 1031 women was 23.9 ± 3 years; mean BMI, 22.5 ± 3.9 kg/m^2^; mean period of gestation, 23.6 ± 7.6 weeks; and mean hemoglobin, 11.2 ± 3.8 gm/dl.

The Pearson’s correlation between VPG and CBG in the fasting state was *r* = 0.433 [intraclass correlation coefficient (ICC) = 0.596, *p* < 0.001], for 1H, it was *r* = 0.653 (ICC = 0.776, *p* < 0.001), and for 2H, it was *r* = 0.784 (ICC = 0.834, *p* < 0.001).

To derive the VPG from the CBG values, the regression equations were fasting VPG = 51.02 +0.36 × fasting CBG; 1-h VPG = 48.91 + 0.52 × 1-h CBG and 2-h VPG = 33.21 + 0.59 × 2-h CBG.

**Fig. 1:**
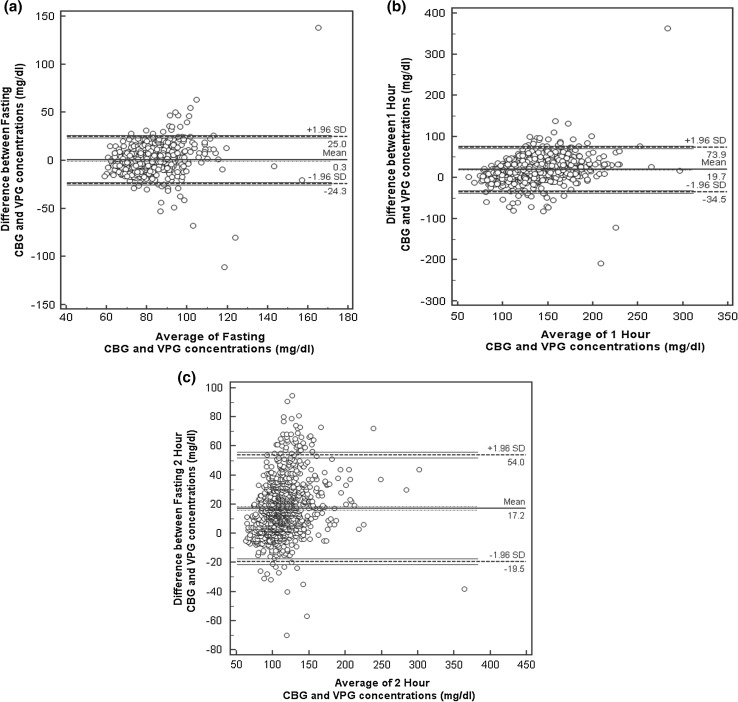
Bland and Altman plots. **a** Fasting CBG and VPG values, **b** 1H CBG and VPG values, **c** 2H CBG and VPG values

Bland–Altman plots were drawn to study the limits of agreement between VPG and CBG for fasting, 1H and 2H cut points (Fig. 1a–c). The mean difference for fasting, 1H and 2H was 0.5, 17.2 and 19.7 mg/dl, respectively, and their 1.96 SD ranged from −20.7 to 21.7 mg/dl, −19.3 to 53.7 mg/dl and −27.6 to 67 mg/dl, respectively.

**Fig. 2:**
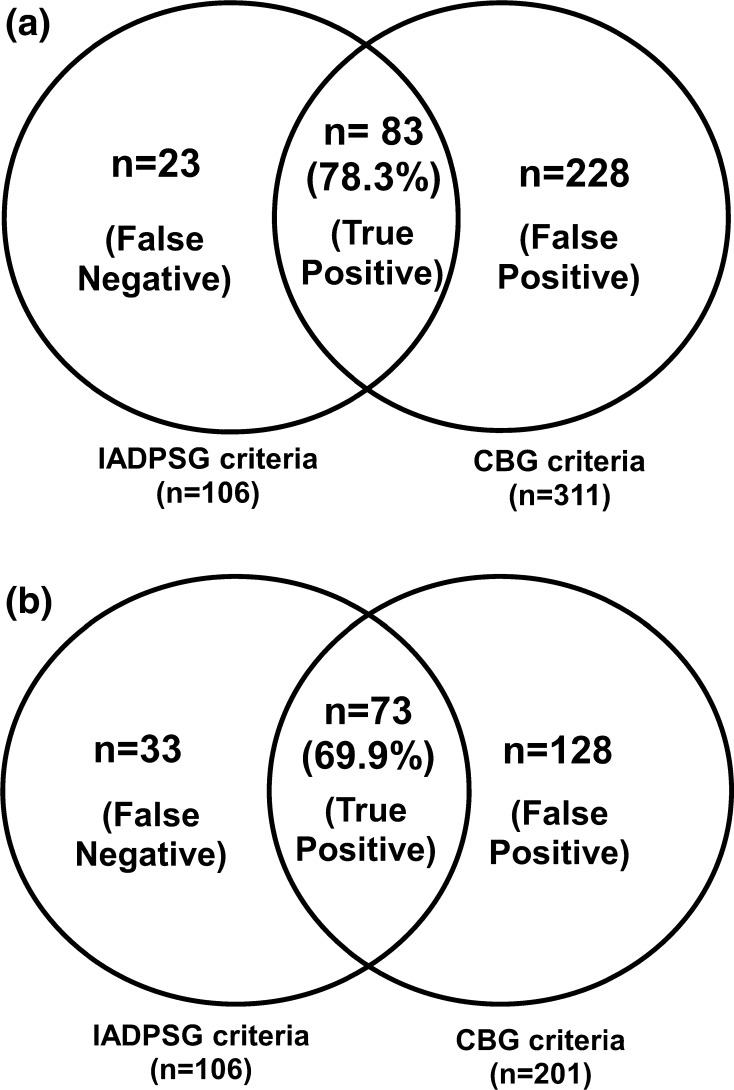
**a** Comparison of diagnosis of GDM by the IADPSG criteria and CBG cut point of Fasting ≥ 92 mg/dl or 1 h ≥ 180 mg/dl or 2 h ≥ 153 mg/dl, **b** comparison of diagnosis of GDM by the IADPSG criteria and CBG cut point of Fasting ≥ 92 mg/dl or 1 h ≥ 200 mg/dl or 2 h ≥ 173 mg/dl

Figure 2a shows the comparison between the IADPSG criteria and the same CBG cut points, i.e., fasting ≥ 92 mg/dl or 1 h ≥ 180 mg/dl or 2 h ≥ 153 mg/dl. Out of the 106 GDM women picked up by the IADPSG VPG criteria, CBG identified 83 (78.3 %). However, additionally 228 women who did not have GDM by IADPSG VPG criteria were identified as having GDM by CBG.

Figure 2b shows the comparison between the IADPSG criteria and the 20 mg/dl higher CBG cut points for the post-stimulated state, i.e., fasting ≥ 92 mg/dl or 1 h ≥ 200 mg/dl or 2 h ≥ 173 mg/dl. Out of the 106 GDM women picked up by the IADPSG criteria, CBG identified 73 women (68.9 %). However, 128 women who did not have GDM by IADPSG criteria were identified as having GDM by CBG.

With the idea of maximizing the sensitivity of a single 2H CBG value, if used as an initial screening tool for GDM, we next constructed receiver operating curves with different 2H CBG cut points and compared the sensitivity, specificity, positive predictive value, negative predictive value and accuracy of these cut points using the IADPSG criteria as the gold standard, and the data are presented in Table 1.

**Table 1:** Comparison of sensitivity and specificity of different 2H CBG levels for the diagnosis of GDM using the IADPSG criteria as the gold standard

2H CBG cut point (mg/dl)	Sensitivity (%)	Specificity (%)	PPV (%)	NPV (%)	Accuracy (%)	% of population who have levels above this value
100 (5.5 mmol/l)	96.2	18.8	12.0	97.8	82.9	82.7
110 (6.1 mmol/l)	92.5	33.7	13.8	97.5	74.2	68.9
120 (6.6 mmol/l)	78.3	53.1	16.1	95.5	65.7	50.1
126 (7.0 mmol/l)	70.8	63.0	18.0	95.0	66.2	40.4
130 (7.2 mmol/l)	67.0	68.7	19.7	94.8	68.1	35.0
140 (7.7 mmol/l)	62.3	80.7	26.3	94.9	76.3	23.7
150 (8.3 mmol/l)	51.9	88.4	34.0	94.1	82.7	15.7
160 (8.8 mmol/l)	40.0	93.9	48.6	91.6	88.3	10.3

A 2H CBG cut point of 126 mg/dl (7.0 mmol/l) gave the optimum sensitivity and specificity of 70.8 and 63 %, respectively, with a C statistic (AUC) of 0.778 (95 % confidence interval, CI 0.726–0.829). If the cut point is lowered to 120 mg/dl (6.6 mmol/l), the sensitivity improved to 78.3 %, and if lowered to 110 md/dl (6.1 mmol/l), it improved to 92.5 %. However, 50.1 and 68.9 % of women, respectively, have to be referred for the definitive diagnostic OGTT using VPG.

Supplemental Table 1 presents the sensitivity and specificity of the fasting capillary blood glucose (fasting CBG) in comparing to the IADPSG. It can be seen that a fasting CBG cut point of 80 mg/dl (4.4 mmol/l) gave the optimum sensitivity and specificity of 67.9 and 58.5 %, respectively, with a C statistic (AUC) of 0.727 (95 % confidence interval, CI 0.673–0.780). For lower cut points of 75 and 70 mg/dl, the sensitivity was 79.3 and 92.5 %, but the corresponding specificity was 36.9 and 16.1 %, respectively. Thus, it is clear that the sensitivity and specificity of the fasting CBG is not satisfactory.

## Discussion

There is considerable controversy regarding the best screening and diagnostic test for diagnosis of GDM. Based on the Hyperglycemia and Adverse Pregnancy Outcomes (HAPO) study, the IADPSG guidelines were introduced in an attempt to reach a global consensus for the diagnosis of GDM [5]. The WHO has recently approved the IADPSG criteria [10], effectively replacing the original WHO 1999 criteria [11] with the IADPSG criteria. However, the WHO 1999 criteria which require only one blood sample (2 h after a 75 g glucose load) are quite widely used in India and other developing countries and even in many developed countries like the UK, because of its simplicity [12]. Nevertheless, both the IADPSG and the WHO 1999 criteria require venous plasma samples which are often a challenge in resource-constrained settings in developing countries.

This paper looks at an alternative method of screening for GDM in resource-constrained settings by means of a handheld glucose meter using CBG. The advantage of using CBG is that even lay people can be trained to do the screening as it serves as a portable, point of care and cost-effective tool for screening. Moreover, obtaining a finger prick sample is minimally invasive when compared with venous blood draw and hence more acceptable to the subjects [13].

Earlier, glucose meters were exclusively used for home glucose monitoring by people with diabetes, but they are gaining acceptance as a screening tool in large epidemiological studies in developing countries such as India due to logistic difficulties in getting venous blood samples due to lack of phlebotomists and lack of standardized laboratories especially in rural areas where 72 % of the population lives [2, 14].

For non-pregnant adults, the WHO has suggested use of the same cut points for CBG as for VPG for diagnosis of diabetes in the fasting state but to use 20 mg/dl (1.1 mmol/l) higher cut points for the 2 h post-glucose value in OGTT [10]. Unfortunately, no such guidelines exist for use of CBG during pregnancy, i.e., for diagnosis of GDM. Hence, we used 2 cut points, a CBG 2H cut point of 140 mg/dl (7.8 mmol/l) which is the 2H VPG cut point for the old WHO 1999 criteria for GDM [11] as well as the 160 mg/dl (8.9 mmol/l) 2H CBG cut point. In addition, we also used the same CBG cut points as the fasting, 1H and 2H VPG IADPSG criteria and also the 20 mg/dl higher cut points for the 1H and 2H CBG values following the WHO recommendations of using 20 mg/dl higher cut points in the non-pregnant state for the stimulated glucose values. We have also derived regression equations for venous plasma glucose in the fasting, 1- and 2-h samples using CBG which can help in calculating the approximate VPG values from CBG measurements.

We found that if we use the same CBG cut points as the IADPSG criteria, 23 women (21.7 %) would be missed, while 33 women (31.1 %) would be missed if CBG cut points of fasting ≥ 92 mg/dl or 1 h ≥ 200 mg/dl or 2 h ≥ 173 mg/dl is used. Moreover, there are a significant number of false positives. So, it is clear that CBG cannot be used for diagnosis of GDM but perhaps can be used as an initial screening test in resource-constrained settings.

Dillon et al. [15] report that CBG values are higher when compared to venous sample due to slow metabolization of glucose in peripheral tissues. Several factors influence the CBG measurements performed by different methods [16–18]. Arterial blood shows higher glucose values than venous blood. The difference may be due to the balance between the water and glucose in the analyzed blood [19] and also due to different glucose meters used in various studies. Factors such as environmental exposure (e.g., moisture, altitude) can affect the accuracy of glucose meter results. Therefore, adequate training should be imparted to the field staff to perform the CBG test correctly. Clinical differences may also alter the accuracy of glucose meters. Conditions affecting blood, such as anemia, have also been documented to alter the settings where such conditions are found to have a higher prevalence. However, there was no difference in the prevalence of anemia between the GDM and the non-GDM women. Hence, this is unlikely to have affected the results of this study.

Based on our results, we conclude that VPG would still need to be used as the diagnostic test for GDM. The sensitivity and specificity of the fasting CBG are not acceptable. However, perhaps the 2H CBG test could be used as an initial screening test in field settings in remote areas where getting venous samples is impossible. All those with values above a certain cut point could be referred to a higher center for a diagnostic OGTT using VPG. Admittedly, the sensitivity for diagnosing GDM using the IADPSG criteria is still not optimum. This is largely because the IADPSG criteria include a fairly low fasting plasma glucose (FPG) cut point of 92 mg/dl (5.1 mmol/l). If a 2H CBG cut point of 120 mg/dl (6.6 mmol/l) or 110 mg/dl (6.1 mmol/l) was used as an initial screening test, the sensitivity improves to 78.3 and 92.5 %, respectively. However, 50.1 and 68.9 % of women would have to be referred to higher centers for the diagnostic OGTT.

While there are little data on the comparative costs of VPG and CBG, one study suggests that doing CBG could help reduce the cost by 80 % compared to VPG done in a laboratory [20]. This could be because of cost of Vacutainers or blood collection tubes, syringes, transportation cost, salaries of laboratory personnel, use of auto analyzers or other laboratory equipments and reagents can be avoided.

Moreover, doing a 2H CBG as an initial screening test might prove to be cost-effective since only a fraction of women would have to be referred for the diagnostic OGTT. Furthermore, even if it is not cost- effective, this may be the only option available in resource-constrained settings where VPG cannot be done. Having said this, proper cost-effective studies need to be done which is one of the limitations of this study. Also, the reliability of glucose meter can be influenced by several environmental factors like humidity. Nevertheless, rigorous training for technicians and continuous quality control measures ensured accuracy of the results. The strength of the study is that it has a fairly large sample size and has systematically compared CBG with the IADPSG criteria which was taken as the gold standard.

In summary, for screening and diagnosing GDM, the VPG still remains the gold standard. However, in resource-constrained situations in developing countries where obtaining venous samples is impossible, the initial screening can be done by CBG using handheld glucose meters with lower 2H glucose cut points (the actual cut points used depending on the resources available) to maximize the sensitivity. Those who screen positive can be referred to higher centers for the definitive diagnostic test using VPG where the gold standard test would be the IADPSG criteria, as it is now being widely accepted worldwide [11, 21].

## Electronic supplementary material

Supplementary material 1 (DOCX 12 kb)
