# Supplementary material for: Use of capillary blood glucose for screening for gestational diabetes mellitus in resource-constrained settings
Source: Acta Diabetol. 2015 Apr 28;53:91–7. doi: 10.1007/s00592-015-0761-9 (PMC4749644; doi:10.1007/s00592-015-0761-9)
Supplement: Supplementary file 1 — Supplementary material 1 (DOCX 12 kb) [file 592_2015_761_MOESM1_ESM.docx]

**SUPPLEMENTAL TABLE 1: Comparison of sensitivity and specificity of different fasting CBG levels for the diagnosis of GDM using the IADPSG criteria as the gold standard**

| **Fasting CBG cut point (mg/dl)** | **Sensitivity (%)** | **Specificity (%)** | **PPV**  **(%)** | **NPV (%)** | **Accuracy  (%)** | **% of population who have levels above this value** |
| --- | --- | --- | --- | --- | --- | --- |
| 60  (3.3 mmol/l) | 100 | 0.8 | 10.4 | 100 | 99.0 | 99.0 |
| 65  (3.6 mmol/l) | 97.2 | 5.1 | 10.5 | 94 | 92.5 | 94.9 |
| 70  (3.8 mmol/l) | 92.5 | 16.1 | 11.2 | 94.9 | 80.7 | 84.6 |
| 75  (4.1 mmol/l) | 79.3 | 36.9 | 12.6 | 93.9 | 64.3 | 64.6 |
| 80  (4.4 mmol/l) | 67.9 | 58.5 | 15.8 | 94.1 | 62.6 | 44.1 |
| 85  (4.7 mmol/l) | 59.4 | 76.7 | 22.7 | 94.3 | 72.0 | 27.0 |
| 90  (4.9 mmol/l) | 47.2 | 88.4 | 31.8 | 93.6 | 82.1 | 15.2 |
| 95  (5.2 mmol/l) | 35.9 | 93.1 | 37.3 | 92.7 | 87.4 | 9.9 |
| 100  (5.5 mmol/l) | 25.5 | 96.3 | 44.3 | 91.8 | 92.1 | 5.9 |
| 105  (5.8 mmol/l) | 18.9 | 97.4 | 45.5 | 91.3 | 94.0 | 4.3 |
| 110  (6.1 mmol/l) | 15.1 | 98.2 | 48.5 | 91 | 95.5 | 3.2 |
